# Supplementary material for: Multi-Detection Size Exclusion Chromatography as an Advanced Tool for Monitoring Enzyme–Antibody Conjugation Reaction and Quality Control of a Final Product
Source: Molecules. 2023 Jun 5;28(11):4567. doi: 10.3390/molecules28114567 (PMC10254844; doi:10.3390/molecules28114567)
Supplement: Supplementary file 1 [file molecules-28-04567-s001.zip › molecules-2407255-supplementary.pdf]

Supplementary Materials

Multi-Detection Size Exclusion Chromatography as an Advanced Tool for Monitoring Enzyme–Antibody Conjugation Reaction and Quality Control of a Final Product

Adela Štimac [1,2,*], Tihana Kurtović [1,2] and Beata Halassy [1,2]

[1] Centre for Research and Knowledge Transfer in Biotechnology, University of Zagreb, Rockefellerova 10, 10000 Zagreb, Croatia
[2] Center of Excellence for Virus Immunology and Vaccines, 10000 Zagreb, Croatia
[*] Correspondence: adela.stimac@unizg.hr

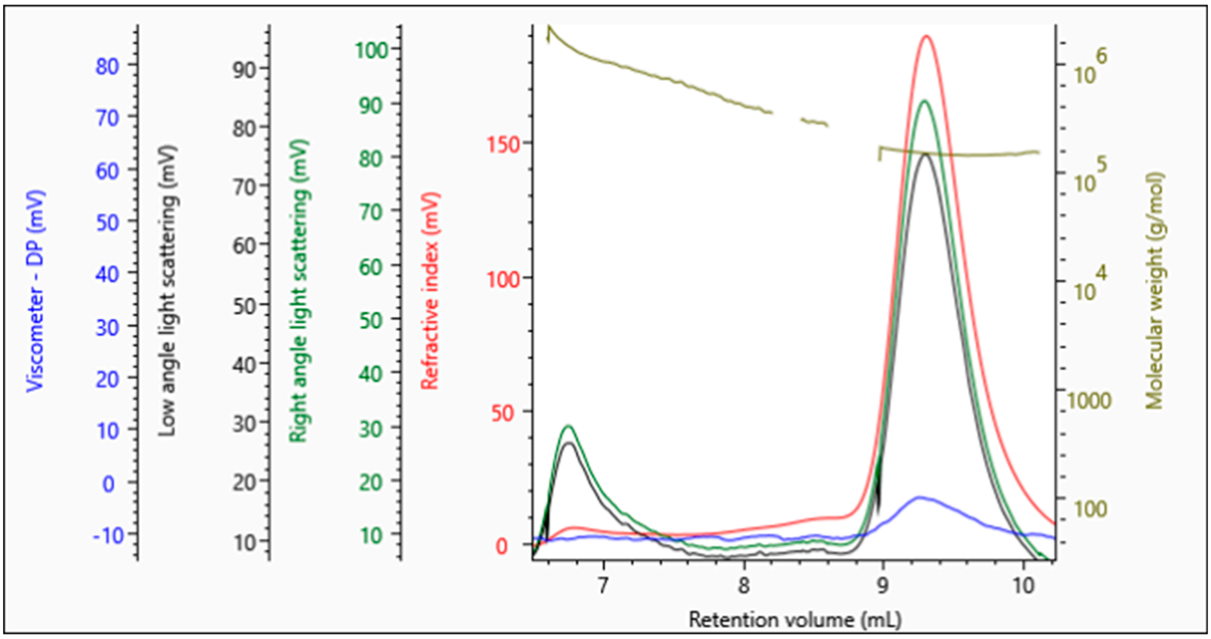

Figure S1. Representative multi-detection SEC chromatograms of IgG sample in phosphate buffer—refractive index (red), right-angle light scattering (green), low-angle light scattering (black), and viscometer (blue). The molecular weight of each species is shown in olive.

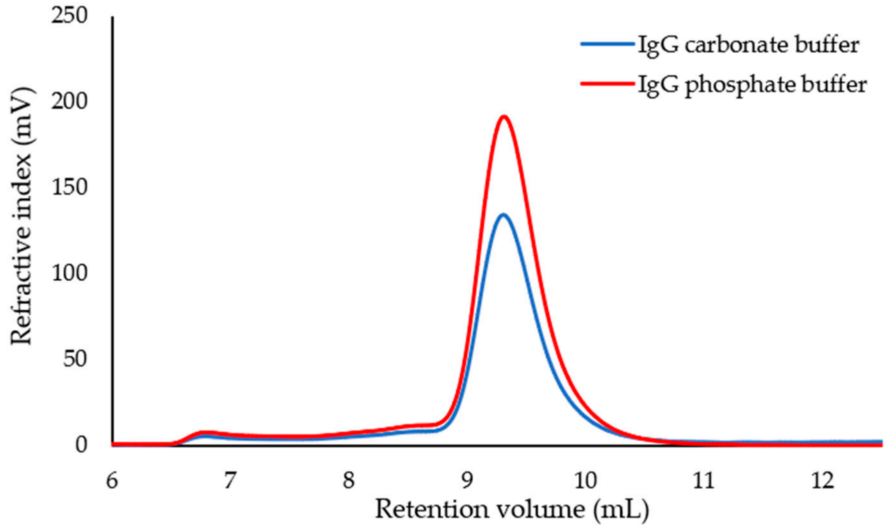

Figure S2. Overlay of RI data obtained for the representative IgG sample in phosphate and carbonate buffer by multi-detection SEC.

Table S1. Summary of the results from multi-detection SEC analysis of IgG samples in phosphate and carbonate buffer. The results are expressed as mean value ± standard error (SE) from *n* measurements.

	IgG in phosphate buffer (*n* = 2)			IgG in carbonate buffer (*n* = 3)		
V_R (mL)	8.2	8.6	9.3	8.2	8.6	9.3
Mw (kDa)	835.7 ± 17.7	300.3 ± 2.2	153.4 ± 0.4	930.7 ± 13.3	309.2 ± 5.1	152.5 ± 1.2
Mw/*Mn*	1.261 ± 0.007	1.002 ± 0.004	1.002 ± 0.000	1.287 ± 0.009	1.002 ± 0.001	1.003 ± 0.000
Share (%)	5.68 ± 1.19	2.12 ± 0.51	92.25 ± 0.67	6.09 ± 0.52	1.87 ± 0.21	92.04 ± 0.21
Identity	higher-order and aggregate	dimer	monomer	higher-order and aggregate	dimer	monomer

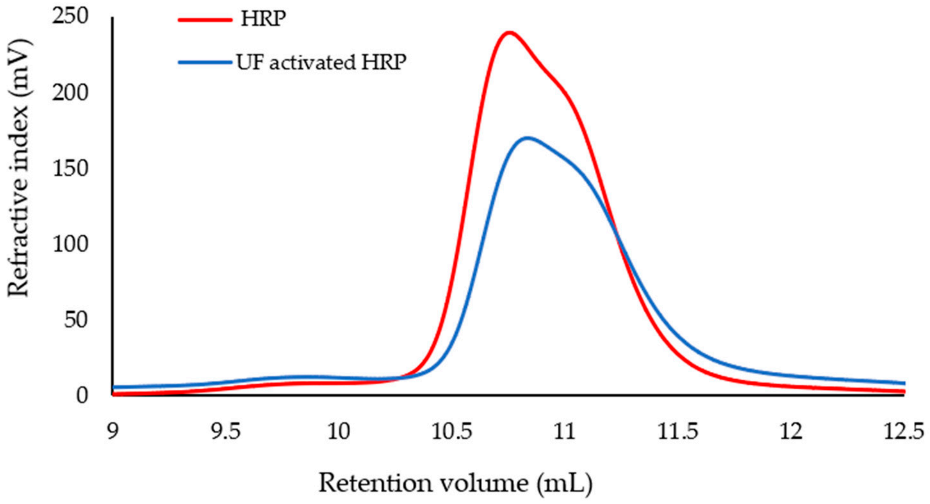

Figure S3. Overlay of RI data obtained for the representative HRP sample before and after activation by multi-detection SEC.

Table S2. Summary of the results from multi-detection SEC analysis of the HRP samples before and after activation. The results are expressed as mean value ± standard error (SE) from *n* measurements.

	HRP before activation (*n* = 3)		UF HRP after activation (*n* = 4)	
V_R (mL)	9.9	10.9	9.9	10.9
Mw (kDa)	73.3 ± 2.4	42.6 ± 0.1	80.5 ± 1.0	43.1 ± 0.5
Mw/*Mn*	1.009 ± 0.004	1.005 ± 0.000	1.010 ± 0.001	1.004 ± 0.001
Rh (nm)	4.4 ± 0.1	2.8 ± 0.0	5.2 ± 0.2	2.8 ± 0.0
Share (%)	4.43 ± 1.03	95.57 ± 0.92	6.21 ± 0.76	93.81 ± 0.72
Identity	n/a	monomer	n/a	monomer

Table S3. Absorbance readings from the ELISA assay obtained for two different concentrations of anti-Vero IgG (1 μg/mL and 10 μg/mL) and various dilutions of anti-Vero IgG-HRP conjugate. A lysate of Vero cells of known concentration was applied as a standard in two-fold serial dilutions, starting from 30 ng/μL.

	Absorbance	
dilution of anti-Vero IgG-HRP conjugate	1 μg/mL anti-Vero IgG	10 μg/mL anti-Vero IgG
1:1000	1.233	2.854
1:2000	0.825	2.038
1:4000	0.566	1.416
1:8000	0.483	1.004
1:16000	0.393	0.674
1:32000	0.353	0.587

A)

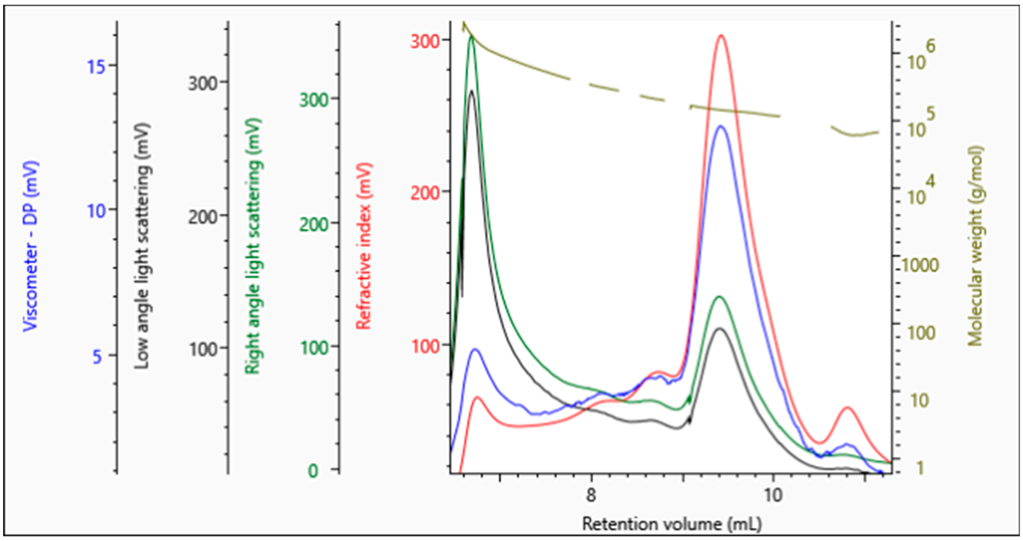

B)

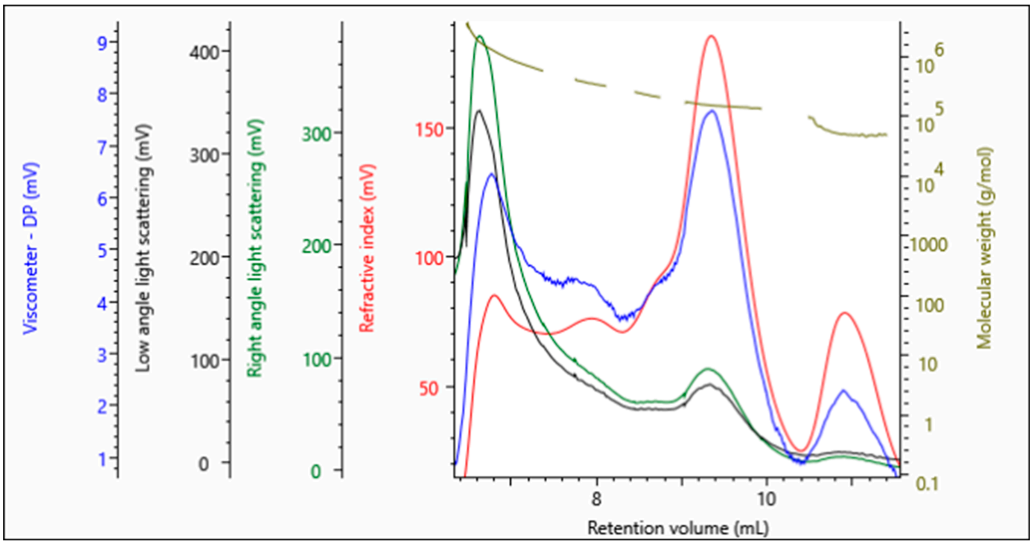

C)

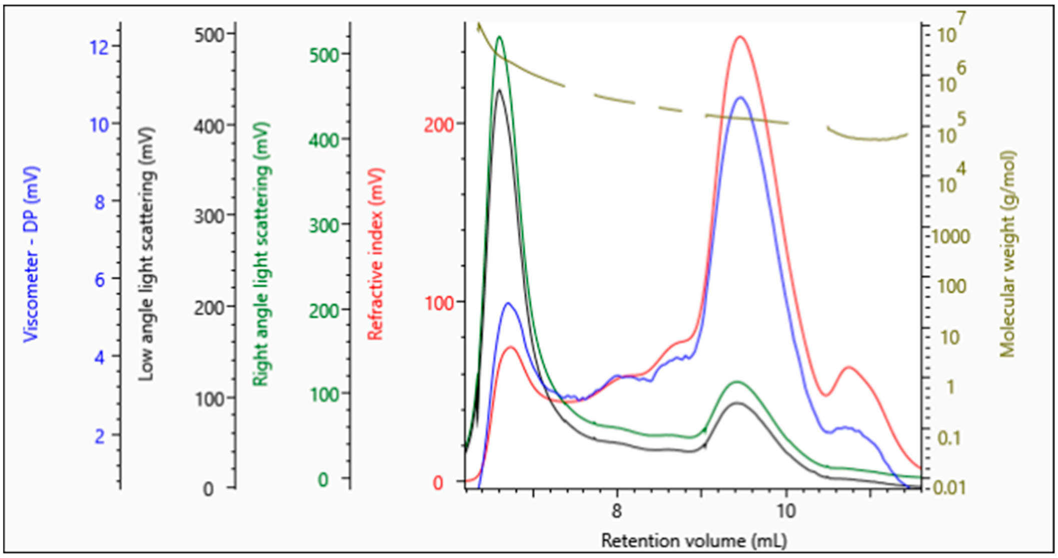

Figure S4. Representative multi-detection SEC chromatograms of commercially available IgG-HRP conjugates: A) anti-goat IgG-HRP produced in rabbit (IgG-HRP 1), B) anti-rabbit IgG-HRP produced in goat (IgG-HRP 2), and C) anti-guinea pig IgG-HRP produced in rabbit (IgG-HRP 3).
